# A trial of intermittent preventive treatment and home-based management of malaria in a rural area of The Gambia

**DOI:** 10.1186/1475-2875-10-2

**Published:** 2011-01-07

**Authors:** Sanie Sesay, Paul Milligan, Ensa Touray, Maimuna Sowe, Emily L Webb, Brian M Greenwood, Kalifa A Bojang

**Affiliations:** 1 Medical Research Council Laboratories, P.O. Box 273, Banjul, The Gambia; 2 London School of Hygiene and Tropical Medicine, London, UK

## Abstract

**Background:**

Individual malaria interventions provide only partial protection in most epidemiological situations. Thus, there is a need to investigate whether combining interventions provides added benefit in reducing mortality and morbidity from malaria. The potential benefits of combining IPT in children (IPTc) with home management of malaria (HMM) was investigated.

**Methods:**

During the 2008 malaria transmission season, 1,277 children under five years of age resident in villages within the rural Farafenni demographic surveillance system (DSS) in North Bank Region, The Gambia were randomized to receive monthly IPTc with a single dose of sulphadoxine/pyrimethamine (SP) plus three doses of amodiaquine (AQ) or SP and AQ placebos given by village health workers (VHWs) on three occasions during the months of September, October and November, in a double-blind trial. Children in all study villages who developed an acute febrile illness suggestive of malaria were treated by VHWs who had been taught how to manage malaria with artemether-lumefantrine (Coartem™). The primary aims of the project were to determine whether IPTc added significant benefit to HMM and whether VHWs could effectively combine the delivery of both interventions.

**Results:**

The incidence of clinical attacks of malaria was very low in both study groups. The incidence rate of malaria in children who received IPTc was 0.44 clinical attacks per 1,000 child months at risk while that for control children was 1.32 per 1,000 child months at risk, a protective efficacy of 66% (95% CI -23% to 96%; p = 0.35). The mean (standard deviation) haemoglobin concentration at the end of the malaria transmission season was similar in the two treatment groups: 10.2 (1.6) g/dL in the IPTc group compared to 10.3 (1.5) g/dL in the placebo group. Coverage with IPTc was high, with 94% of children receiving all three treatments during the study period.

**Conclusion:**

Due to the very low incidence of malaria, no firm conclusion can be drawn on the added benefit of IPTc in preventing clinical episodes of malaria among children who had access to HMM in The Gambia. However, the study showed that VHWs can successfully combine provision of HMM with provision of IPTc.

**Trial Registration:**

ClinicalTrials.gov NCT00944840

## Background

Although the incidence of malaria appears to be declining in a number of African countries, it remains an important cause of mortality and morbidity among young children and pregnant women. Malaria control strategies deployed in Africa include prompt treatment of clinical attacks of malaria with an effective anti-malarial drug combination, vector control using insecticide-treated nets (ITNs) or curtains or indoor residual spraying (IRS) and intermittent preventive treatment (IPT). However, individually these interventions provide only partial protection in most epidemiological situations [1]. Thus, there is a need to investigate whether combining interventions provides added benefit in reducing mortality and morbidity.

Studies have shown that IPT with sulphadoxine/pyrimethamine (SP) given on two or three occasions during pregnancy provides significant protection against maternal anaemia [2] and low birth weight [3], especially in primigravidae and secundigravidae in whom the risk of malaria is highest. These encouraging results generated interest in the use of IPT for the prevention of malaria and malaria-associated severe anaemia in infants and children. IPT with SP given at the time of routine infant immunization (IPTi) reduces the incidence of malaria in the first year of life by about 30% [4] and IPTi is now recommended for introduction as a malaria control strategy in countries where there is a significant burden of malaria in infants and where SP resistance is low [5]. However, in many parts of Africa, in particular those within the Sahelian and sub-Sahelian regions where malaria transmission is seasonal, the main burden of malaria is not in infants but in older children [6-8]. The potential for IPT to prevent malaria and anaemia in children over the age of one year has been evaluated in a limited number of trials carried out mainly in West Africa. Results from these trials have indicated that IPT in children aged one to five years (IPTc) provided between 40% to 86% protection against clinical attacks of malaria [9-11]. These results suggest that IPTc has potential as an affordable malaria and anaemia control measure.

Home-based management of malaria (HMM) is now considered an important strategy for improving treatment practices [12,13] and for reducing severe morbidity and mortality from malaria in resource-poor countries [14,15]. HMM involves presumptive treatment of febrile children at or near home with anti-malarial drugs distributed by trained members of the community [16]. Community distributors provide medications and educate primary caregivers about treatment of malaria, administration of anti-malarial drugs, and recognition of severe illness. A recent systemic review concluded that the impact of HMM on morbidity and mortality endpoints has been mixed [17]. Two studies showed no health impact, while another showed a decrease in malaria prevalence and incidence, but no impact on mortality. One study in Burkina Faso suggested that HMM decreased the proportion of severe malaria cases, while another study from the same country showed a decrease in the risk of progression to severe malaria. Of the four studies with mortality endpoints only one, from Ethiopia, showed a positive impact, with a reduction in the under-5 mortality rate of 40.6% (95% CI 29.2% - 50.6%)[17].

Although a number of trials of IPTc and HMM in children, given as individual interventions, have been conducted, only one trial has formally addressed the added value of IPTc in children who have access to HMM. In this study conducted in Ghana, IPTc with amodiaquine and artesunate was given three times during the course of the year to children aged 6 - 60 months who also had access to HMM. The prevalence of malaria parasitaemia was significantly lower at the end of the intervention than at baseline but, as no control group was included in the study, it is not possible to define the exact role played by IPTc in achieving this improvement [18]. An additional placebo controlled trial to compare HMM with Coartem™ combined with IPTc to HMM with Coartem™ alone in reducing morbidity from malaria in an area of The Gambia with seasonal transmission of malaria has been undertaken.

## Methods

### Study area and population

The study, based at the MRC Field Station, Farafernni in the North Bank Region of The Gambia, was conducted between May 2008 and January 2009. The trial was undertaken in a group of 24 villages near Farafenni which are part of the rural Farafenni demographic surveillance system (DSS), described in detail elsewhere [19]. There are 18 rural primary health care (PHC) villages in the study area, two health centres and one hospital in the study area. In PHC villages, village health workers (VHWs) are given six to eight weeks of training on how to treat common conditions such as uncomplicated malaria, acute respiratory infections, diarrhoeal diseases and minor injuries. Since 2008, the first-line treatment for uncomplicated malaria has been artemether- lumefantrine (Coartem™, Novartis Pharma, Basel Switzerland).

Malaria in The Gambia occurs almost exclusively during the rainy season and immediately afterwards (July to November) with peak transmission occurring during October and November. The annual entomological inoculation rate varies enormously across the country and estimates have been reported in the range of 1-177 [20].

### Study design

The study was designed as a double-blind, randomized placebo controlled trial. Children were individually randomized to receive monthly treatment with a single dose of SP plus three doses of amodiaquine (AQ) or SP and AQ placebos from a VHW at monthly intervals on three occasions during the months of September, October and November. All study children had ready access to a VHW who had been trained to administer HMM with Coartem™ to children who developed symptoms compatible with malaria during the malaria transmission season.

### Screening and enrolment

Prior to the start of the trial, meetings were held in all potential study villages to explain the purpose and methods of the study and to answer questions from parents and guardians of eligible children. During these meetings, agreement of a village to participate in the study was obtained from the villagers. A list of children living in the 24 villages, which agreed to join the study who would be aged between six and 59 months at the time of the first treatment with IPTc in September 2008 was obtained from the DSS database. These children and their parents were invited for screening for potential participation in the trial between July and August 2008. Written informed consent was obtained from parents or guardians of all potentially eligible children before screening. The age and identity of these children were checked, and they were examined to rule out any clinically significant disease that might interfere with the outcome of the trial. Children were considered eligible if they had no clinically significant acute or chronic disease. Exclusion criteria included known allergy to any anti-malarial drug or the presence of acute or chronic, clinically significant pulmonary, cardiovascular, hepatic or renal disease. A finger-prick blood sample was collected from all children who met the inclusion criteria for preparation of a thick blood smear and measurement of haemoglobin (Hb) concentration. If a child had symptoms suggestive of malaria, the blood film was examined shortly after collection, whilst remaining blood films were examined at a later date. Children with acute malaria were treated with Coartem™ and excluded from the study and those with anaemia were treated with oral iron.

A reporter based in each village was asked to keep records of all deaths, births and immigrations and emigrations that occurred during the study period. Village reporters were visited fortnightly by a project field worker and their records collected and checked. Deaths were investigated using a modified version of the INDEPTH post mortem questionnaire [21].

### Randomization and drug distribution

One thousand two hundred and seventy-seven eligible children whose parents consented were individually randomized into either the SP plus AQ or placebo group in a 1:1 ratio. The randomization list was generated using permuted blocks of 12 by the trial statistician, using the random number generator of STATA version 10. One tablet of SP plus three tablets of AQ or one tablet of SP placebo plus three tablets of AQ placebo were placed in sealed opaque envelopes labelled with the randomization number. For each study subject, a set of three envelopes with the same randomization number, one for each month were prepared. Each child enrolled was assigned the next study number in sequence. Neither the VHWs who administered the treatment, the field or laboratory staff who collected data nor the families of study children knew which was the active drug and which placebo.

A recording system for drug administration that could be used by VHWs, many of whom were illiterate, was devised. This consisted of a treatment card held by the mother or guardian of each child and an IPT register held by the VHW. The treatment card and the IPT register were labelled with the child's, mother's and village's names and compound, demographic surveillance and study numbers. The nature of the treatment and the number of tablets of that treatment that each child should receive was indicated on the treatment card using a colour code to indicate the treatment (white for SP or SP placebo and yellow for AQ or AQ placebo) and full circles, half circles and quarter circles to indicate the number of whole, half or quarter tablets that the child should receive. Dosage was based on the child's age. Children aged 3-11 months received half a tablet of SP or SP placebo and a whole tablet was given to children aged 1-5 years. One quarter, half or whole tablet of AQ or AQ placebo was given daily for three days to children aged 3-11 months, 1-2 years and 3-5 years respectively. SP tablets (500 mg sulphadoxine/25 mg pyrimethamine) and amodiaquine (AQ) tablets (200 mg base tablets) were obtained from Kina Pharma Ltd, Accra, Ghana and their solubility and active drug content confirmed at the London School of Hygiene and Tropical Medicine. The first dose of treatment was given under direct observation, the second and third doses by the parent or guardian at home.

### Morbidity surveillance during the rainy season

Passive surveillance for malaria was carried out during the 2008 transmission season. Parents or guardians were encouraged to take their child to the VHW at any time their child became unwell. VHWs were instructed to presumptively treat febrile illnesses suggestive of malaria with Coartem™.

One field worker was attached to two to three VHWs during the surveillance period. The role of the field worker was to prepare a thick blood smear from children who had been diagnosed as a case of malaria by the VHW and scheduled for treatment with Coartem™ for subsequent confirmation of the diagnosis. Details of children treated by the VHWs were recorded in books with help from field workers. VHWs referred children who failed to improve on treatment and those with danger signs (breathing difficulty, severe weakness, convulsions, severe diarrhoea or vomiting) to the nearest health centre or to the AFPRC Hospital in Farafenni for further evaluation and management. Project staff members were based at each of these health facilities to identify children in the trial when they presented for treatment and to ensure that they were properly investigated and treated promptly. At each clinic visit, axillary temperature was recorded using a digital thermometer and Hb concentration measured using a Hemocue machine. A Rapid Diagnostic Test for malaria (RDT) (Optimal, DiaMed AG, Cressier, Switzerland) was done if a child had fever (axillary temperature of ≥ 37.5°C) or a history of fever within the previous 48 hours. In such cases, a thick blood smear was also collected for subsequent confirmation of the diagnosis. Children with documented fever (axillary temperature of ≥ 37.5°C) or a history of recent fever and malaria parasitaemia or a positive RDT were treated with Coartem™. Children with severe malaria were treated with intramuscular quinine. The treatment of study subjects seen at the health centres for conditions other than malaria was carried out in accordance with national guidelines.

A cross-sectional survey of all available study children was undertaken at the end of the malaria transmission season. Children were examined by a project clinician and anthropometric data were collected and a finger-prick blood sample was obtained for preparation of thick blood smears and determination of Hb concentration.

### Laboratory methods

Thick smears were prepared in duplicate. If a child had symptoms of malaria, one smear was stained with Field's stain and read promptly to guide treatment. The other smear was stained with Giemsa and 200 high power fields (HPF) were examined before a smear was declared negative. Only the Giemsa-stained slide readings were used for the trial analysis. Parasite density was expressed per μl with the assumption that 1 parasite per high-powered field (hpf) equals a density of 500 parasites per μl. All slides were read by two laboratory technicians. If there was disagreement between their readings on parasite positivity or if the difference of the log-densities recorded was more than 1.5, slides were read by a third technician. Agreement was reached among the three technicians after the slides had been re-checked. When a laboratory technician was not immediately available to read a thick blood smear the result of the RDT was used to guide treatment, and a thick blood smear collected for subsequent confirmation of the diagnosis. Hb concentration was measured at recruitment, during morbidity surveillance and at the end of malaria transmission season surveys using a portable haemoglobinometer (HemoCue AB, Sweden).

### Data management

Data from participants were recorded on paper forms and were checked by field supervisors, study physician, and data manager for consistency and accuracy. All data were entered twice into a SQL database using MS Access software. The accuracy of data input was checked and validated using customized validation programmes. The cleaned data were converted to STATA version 10 file (STATA Corporation, Texas, USA) prior to analysis.

### Statistical analysis

The primary study endpoint was the incidence of clinical malaria (documented fever [axillary temp ≥ 37.5°C] or a history of fever within the previous 48 hours accompanied by asexual malaria parasitaemia at a density of ≥5,000 parasites/μL) among study subjects seen at one of the health centres or at the district hospital during the surveillance period. Secondary endpoints included incidence of a febrile illness with parasitaemia at any density among children seen by a VHW or at a health centre or hospital, incidence of anaemia among children seen at a health centre or hospital, the prevalence of parasitaemia at the end of malaria transmission season cross-sectional survey, the prevalence of anaemia at the end of malaria transmission season cross-sectional survey, the proportion of children who received three IPTc treatment courses and the proportion of children who received no IPTc treatment course.

The incidence of malaria seen at a health centre or hospital in children who had access to HMM and who received placebo was estimated to be 0.2 episodes per child per transmission season. To detect a 40% reduction in the incidence of malaria with a power of 90%, using a significance level of 5% and assuming 20% loss to follow-up in children who received IPTc, 625 children per group were needed. For the cross-sectional survey, assuming a parasite prevalence of 20% at the end of transmission season in children who had access to HMM and received placebo, 557 children in each study group were needed to show a 40% reduction in the prevalence of malaria in children who received IPTc in addition to HMM with 90% power at the 5% level of significance assuming a 20% loss to follow-up. A reporting and analysis plan (RAP) was developed prior to breaking the code. The primary analysis included all children who were randomized, regardless of the number of treatments received. Characteristics of all children enrolled in the study were tabulated by study arm. For the primary outcome of malaria incidence during the study period, analysis was by intention to treat. Time at risk was calculated from date of enrolment until the date of cross-sectional survey or until date of death or emigration from the study area with no return. No child had more than one episode of malaria so no adjustment for clustering was required. The protective effect of IPTc in addition to HMM against malaria during the surveillance period was calculated as 100(1-R), where R is the incidence rate ratio for the IPTc versus the placebo group, calculated using Cox regression. The effects of IPTc on incidence of anaemia and other complaints were calculated in the same way.

Prevalences of anaemia and parasitaemia in the cross-sectional survey at the end of the malaria transmission season were compared between the two groups using logistic regression. Hb concentrations at the end of the transmission season were compared using linear regression, adjusting for Hb at enrolment. Coverage with IPTc was evaluated from records kept by VHWs of treatment administration. The proportions of children who had received each monthly treatment course, those who received three treatment courses, and those who received no treatment were tabulated.

### Ethical review

The study was approved by the London School of Hygiene & Tropical Medicine Ethics Committee and by the joint MRC/Gambia Government Ethics Committee.

## Results

### Baseline characteristics

**Figure 1:**
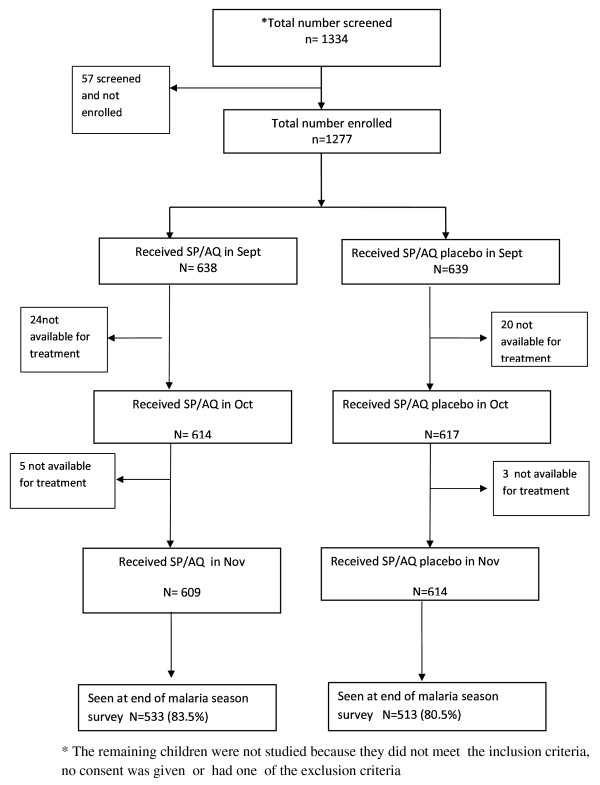
**Trial profile**. * The remaining children were not studied because they did not meet the inclusion criteria, no consent was given or had one of the exclusion criteria

One thousand three hundred and thirty-four children aged 6-59 months were selected from the Farafenni DSS and screened; 1,277 children who met the entry criteria were enrolled and allocated to receive monthly IPT with SP plus AQ (639) or SP placebo plus AQ placebo (638). The remaining 57 were not enrolled because they did not meet the inclusion criteria, no consent was given or because they had one of the exclusion criteria (Figure 1). At enrolment, the two treatment groups were similar with respect to baseline features such as age, ethnic group and mean Hb concentration (Table 1). *Plasmodium falciparum *parasitaemia was found infrequently at enrolment; only 0.5% of study subjects in either treatment group had parasitaemia; 93% of the study subjects slept under an ITN.

**Table 1:** Enrolment characteristics of children in the two study groups

	Intervention group	
Variable	Placebo (n = 638)	SP/AQ (n = 639)
Age (months) (mean, SE)	29.9 (16.5)	30.1 (17.3)

Sex (% male)	323 (51.4%)	309 (49.6%)

Sleep nightly under impregnated bed net	596 (93.3%)	596 (93.3%)

Ethnic group		
Fula	241 (38.0%)	238 (38.0%)
Mandingo	73 (11.5%)	63 (10.1%)
Wollof	311 (49.0%)	316 (50.5%)
Other	20 (1.6%)	9 (1.4%)

Mean haemoglobin g/dl	10.2 (1.4)	10.1 (1.5)

Splenomegaly	16 (3.0%)	18 (3.4%)

Malaria parasitaemia	3 (0.5%)	3 (0.5%)h

### Coverage with IPTc

Table 2 shows the number of children who came for their scheduled monthly treatment during the malaria transmission period. A high rate of coverage was achieved throughout the trial. Among the 1277 study subjects enrolled in the study, 98.8% in each group received treatment 1, 96.1% and 96.7% in the SP/AQ and the control groups respectively received treatment 2, and 95.3% and 96.2% in the SP/AQ and placebo groups respectively received treatment 3. The most common reasons for missing a dose were travelling out of the study area or parental refusal.

**Table 2:** Number of treatment courses of trial medication received

Treatment round	Outcome	Placebo	SP/AQ	
Treatment 1 (September)	Received	637 (99.8%)	638 (99.8%)	
	Refused	0	0	
	Absent	1 (0.2%)	1 (0.2%)	

Treatment 2 (October)	Received	617 (96.7%)	614 (96.1%)	
	Refused	7 (1.1%)	8 (1.3%)	
	Absent	14 (2.2%)	17 (2.7%)	

Treatment 3 (November)	Received	614 (96.2%)	609 (95.3%)	
	Refused	9 (1.4%)	13 (2.0%)	
	Absent	15 (2.4%)	17 (2.7%)	

Overall treatment coverage				p-value

% with 3 treatments		604 (94.7%)	597 (93.4%)	0.35

% with 0 treatments		1 (0.2%)	1 (0.2%)	1.00

### Impact of the interventions on morbidity from malaria and on anaemia

The incidence of clinical attacks of malaria was very low in each treatment group; no child had more than one episode of malaria. There were no cases of malaria with parasitaemia at a density of ≥5,000 parasites/μL. Table 3 shows the number of malaria episodes with any parasitaemia detected during the study among children attending a health centre or hospital, and among children seen by a VHW. During the malaria transmission season only one clinical episode of malaria was recorded at a health centre in children who received SP plus AQ and only two episodes in children who received placebo. One additional episode was detected by a VHW in a child in the placebo group. The incidence rate of malaria detected by any means in children who received the intervention was 0.44 per 1,000 child months at risk while that for control children was 1.32 per 1000 child months at risk, giving a protective efficacy of 66% (95% CI -228% to 96%; p = 0.35). There were no differences in incidence of anaemia or other morbidity indicators during the surveillance period (Table 4).

**Table 3:** Frequency of malaria during the study

Outcome	Placebo			SP/AQ			Protective efficacy (95% CI)	P value
	Events	Person months at risk	Incidence rate/1000 person months	Event	Person months at risk	Incidence Rate/1000 person months		
Clinical malaria, diagnosed at OPD	2	2279	0.88	1	2248	0.44	49% (-468%, 95%)	0.59

Clinical malaria, diagnosed at OPD or by VHW	3	2279	1.32	1	2248	0.44	66% (-228%, 96%)	0.35

**Table 4:** Morbidity in children who received SP plus AQ or placebo during the malaria transmission period

	Placebo (person months at risk, 2279)		SP/AQ (person months at risk, 2248)			
Outcome	Events	Incidence rate/100 person months	Events	Incidence rate/100 person months	Protective efficacy (95% CI)	P
Total outpatient visits	142	6.2	155	6.9	-11% (-42%, 14%)	0.42
Anaemia (Hb < 11 g/dL)	71	3.1	70	3.1	0% (-44%, 30%)	0.99
Moderate anaemia (Hb < 8 g/dL)	14	0.6	13	0.6	6% (-100%, 60%)	0.89
Fever (temp ≥ 37.5°C)	33	1.4	28	1.2	14% (-42%, 48%)	0.55
Upper respiratory tract infection	48	2.1	49	2.2	-4% (-58%, 32%)	0.86
Skin/soft tissue infection	25	1.1	21	0.9	15% (-53%, 47%)	0.59
Gastroenteritis	42	1.8	39	1.7	6% (-60%, 45%)	0.83

One thousand and forty-six study children (513 SP/AQ, 533 placebo) were seen at the end of the malaria transmission season. Three (0.67%) children in the SP/AQ group had asexual stage *P. falciparum *parasitaemia compared with 5 (0.9%) in the control group (p = 0.73). The prevalence of splenomegaly at the end of malaria transmission season was also very low in the two groups (2.0% SP/AQ vs 3.0% placebo; p = 0.29). The proportions of children with a Hb concentration of < 8 g/dL at the end of the malaria transmission season were similar in the two groups of children, 44/513(8.6%) in children who received SP/AQ and 45/533 (8.4%) in those who received placebo (OR adjusted for baseline Hb 0.81, 95% CI 0.48, 1.36; P = 0.41). The mean (standard deviation) Hb concentration at the end of the malaria transmission season was similar in the two treatment groups; 10.2 (1.6) g/dL in the SP/AQ group compared to 10.3 (1.5) in the placebo group, difference adjusted for baseline Hb 0.04 (95% CI -0.09, 0.17; P = 0.55).

### Safety of IPTc with SP/AQ

There was only one death during the surveillance period among the 1,277 study children. This child, who was in the placebo group, died at home in December; a verbal autopsy suggested a diagnosis of pneumonia. Fourteen children were admitted to one of the health facilities in the study area during the surveillance period, two of them twice. The 16 admissions were equally distributed between treatment arms, nine children were diagnosed with pneumonia (five and four in the intervention and control groups, respectively) and with acute gastroenteritis (2 and 1 in the intervention and control groups respectively). Remaining admissions were for anaemia, malnutrition, upper respiratory tract infection and severe helminth infection. One child in the SP/AQ group developed a drug eruption which presented as multiple areas of macular hyperpigmentation of different sizes mainly on the trunk with sharply demarcated outlines.

## Discussion

This study was carried out to determine whether IPTc with SP plus AQ provided additional benefit in reducing malaria morbidity in children who received HMM in the context of high ITN coverage. Very few cases of malaria were detected in either arm of the trial so the study was not able to determine its primary objective. In addition, IPTc with SP plus AQ did not have any measurable impact on anaemia or splenomegaly.

The very low incidence of malaria encountered in the Farafenni area of The Gambia was surprising but consistent with other recent data from The Gambia that have indicated a dramatic fall in the incidence of malaria in The Gambia in recent years [8]. Why such a dramatic decline in malaria incidence has occurred over a relatively short period of time is not clear. Improvements in overall living and educational standards, a change of first line treatment from ineffective chloroquine to SP and then to artemisinin combination therapy (ACT) and scaling up of the distribution of ITNs (coverage of ITN among the study population was about 93%) are all likely to have played a part.

In recent years, significant reductions in the incidence of malaria have been reported in several countries in Africa where malaria was previously moderately endemic [22]. This success in malaria control has led to a renewed interest in the possibility of national or regional elimination of malaria in endemic areas with the ultimate goal of eradicating the infection. Malaria elimination will require the use of combination of interventions and results from this trial highlights the importance of conducting research to determine how combinations of interventions can be used most effectively at different stages of elimination. This trial showed that in an area with seasonal transmission, a currently low incidence of malaria and with high ITN coverage, IPTc did not provide any additional benefit to HMM. However, this might not be the case in other areas where much higher levels of seasonal malaria have persisted, such as parts of Burkina Faso and Mali.

No serious adverse events which could be directly attributed to the study medication were observed. One skin rash was observed but this did not have features of Stevens Johnson syndrome.

IPTc would not be a useful intervention in The Gambia if the incidence of malaria persists at its currently very low level. However, recent studies in Burkina Faso and Mali [23,24] have demonstrated that IPTc with the same drug combination used in this trial reduced the incidence of uncomplicated and severe malaria by 70-80% in children who slept under an ITN. In such epidemiological situations, IPTc may prove to be a very valuable intervention if an effective method of drug delivery can be achieved. In this study we have shown how VHWs can be trained to deliver both IPTc and HMM effectively and how high coverage levels with IPTc can be achieved. A simple delivery system that can be used by community health workers with little training, some of whom were illiterate, was devised. This approach to malaria control needs to be re-evaluated in other areas where malaria transmission remains high and where IPTc could have a major impact.

## Conflicts of interest statement

The authors declare that they have no competing interests.

## Authors' contributions

KAB, BG conceived and designed the experiments. KAB, SS performed the experiments. EW, KAB, PM, SS analysed the data. ET, MS contributed reagents/materials/analysis tools. KAB PM, EW, BG wrote the paper. All authors read and approved the final manuscript.
